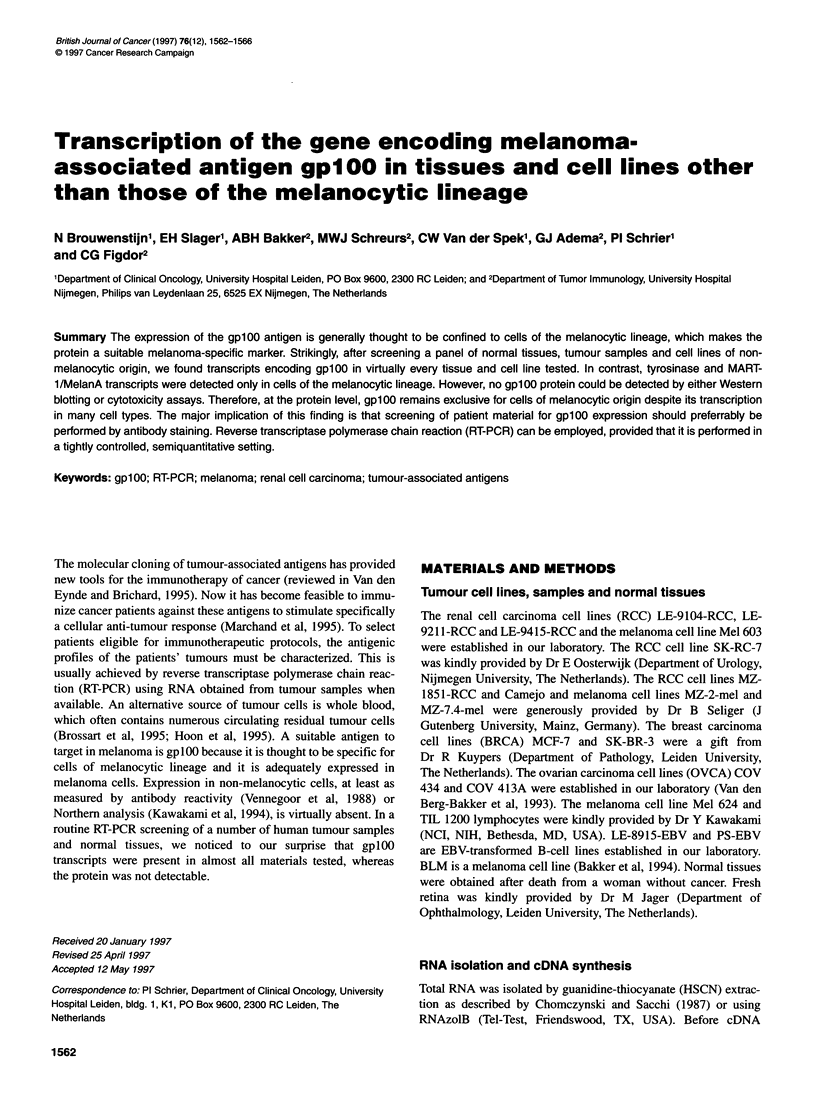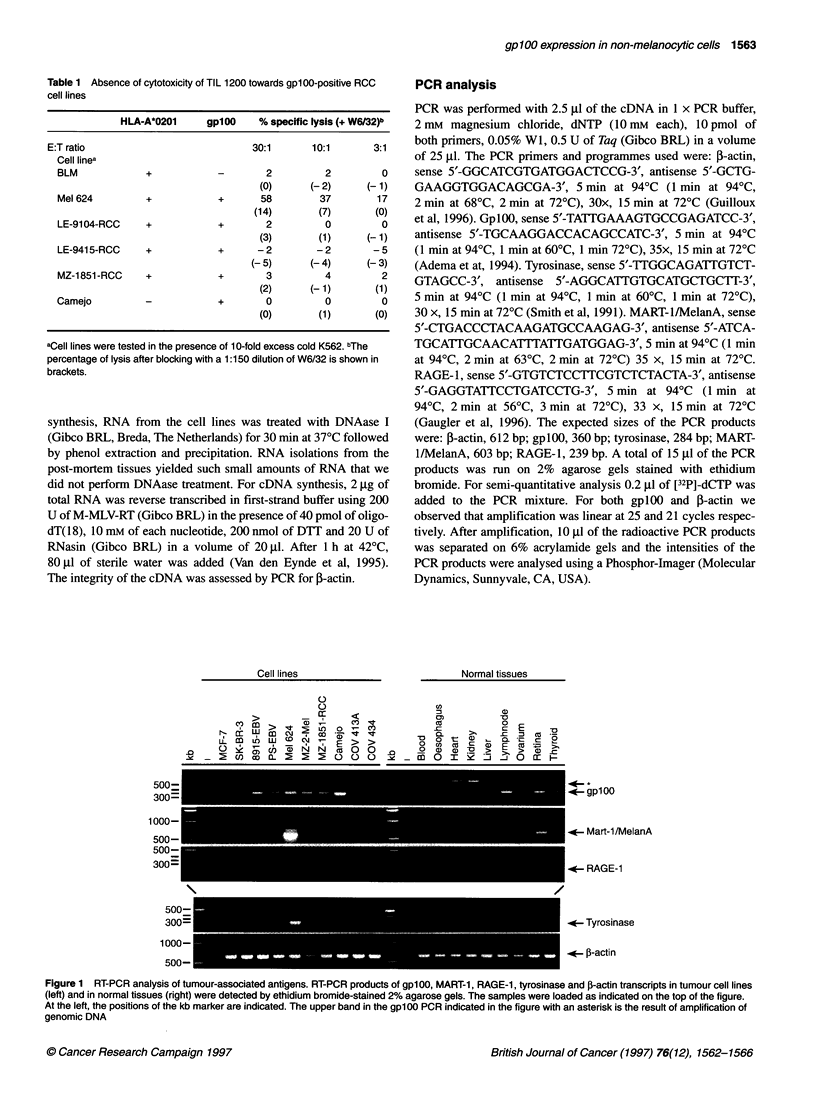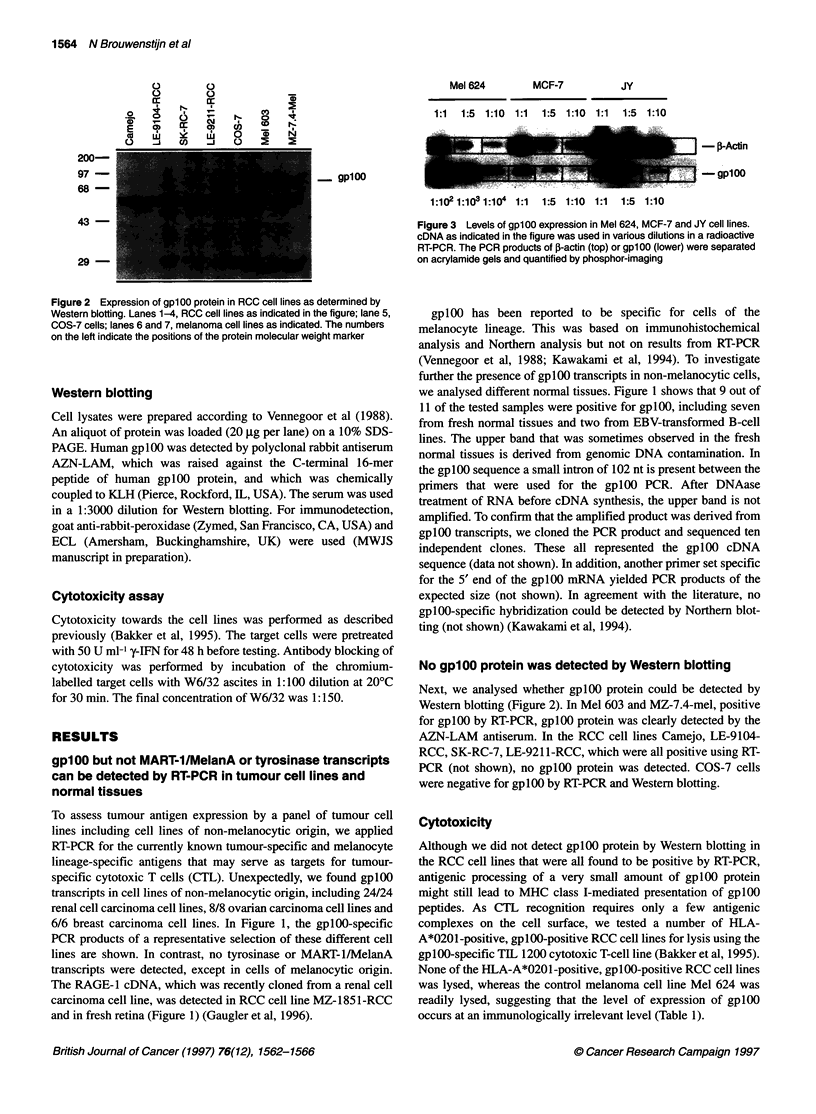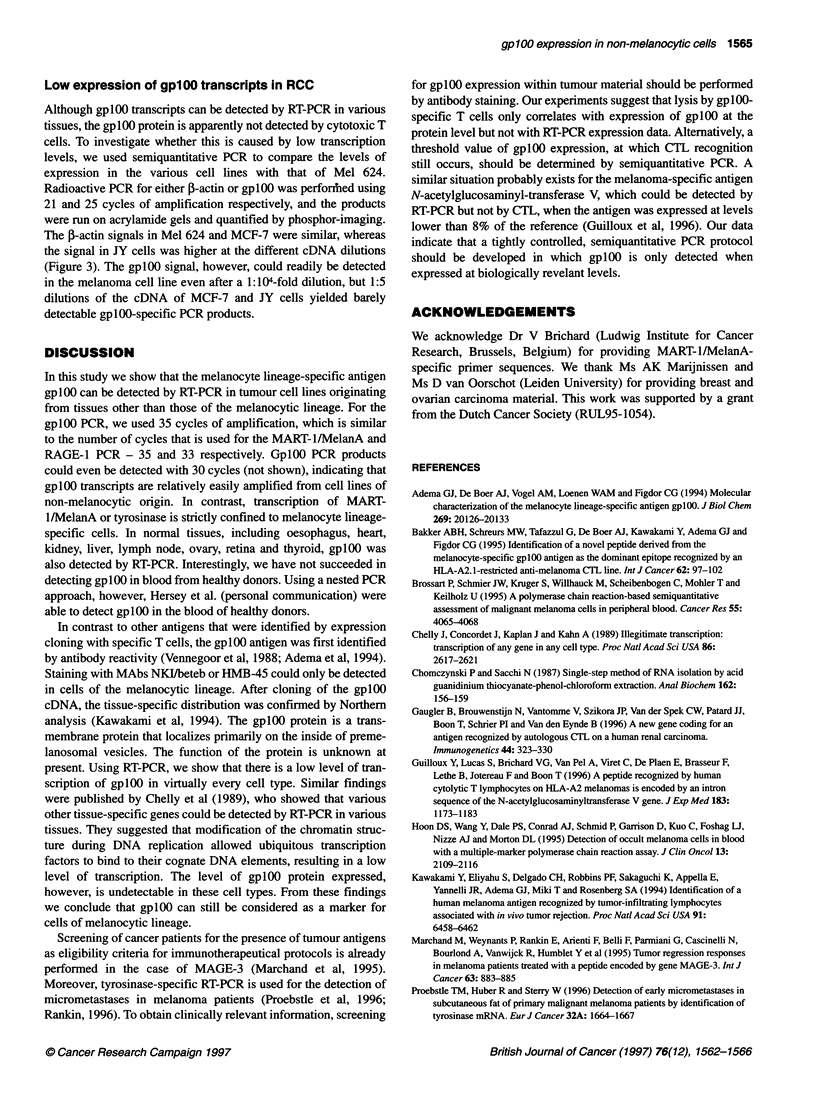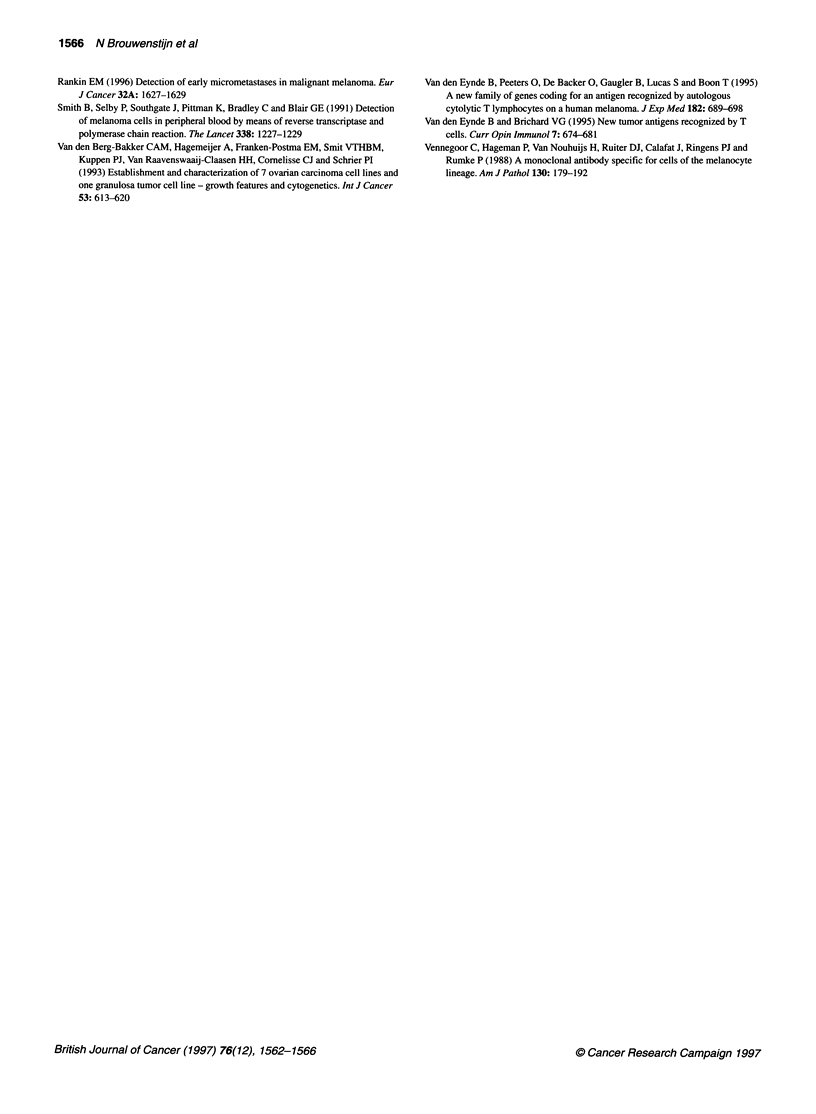# Transcription of the gene encoding melanoma-associated antigen gp100 in tissues and cell lines other than those of the melanocytic lineage.

**DOI:** 10.1038/bjc.1997.597

**Published:** 1997

**Authors:** N. Brouwenstijn, E. H. Slager, A. B. Bakker, M. W. Schreurs, C. W. Van der Spek, G. J. Adema, P. I. Schrier, C. G. Figdor

**Affiliations:** Department of Clinical Oncology, University Hospital Leiden, The Netherlands.

## Abstract

**Images:**


British Journal of Cancer (1997) 76(12), 1562–1566
© 1997 Cancer Research Campaign

### Transcription of the gene encoding melanoma-associated antigen gp100 in tissues and cell lines other than those of the melanocytic lineage

**N Brouwenstijn[1], EH Slager[1], ABH Bakker[2], MWJ Schreurs[2], CW Van der Spek[1], GJ Adema[2], PI Schrier[1] and CG Figdor[2]**

[1]Department of Clinical Oncology, University Hospital Leiden, PO Box 9600, 2300 RC Leiden; and [2]Department of Tumor Immunology, University Hospital Nijmegen, Philips van Leydenlaan 25, 6525 EX Nijmegen, The Netherlands

**Summary** The expression of the gp100 antigen is generally thought to be confined to cells of the melanocytic lineage, which makes the protein a suitable melanoma-specific marker. Strikingly, after screening a panel of normal tissues, tumour samples and cell lines of non-melanocytic origin, we found transcripts encoding gp100 in virtually every tissue and cell line tested. In contrast, tyrosinase and MART-1/MelanA transcripts were detected only in cells of the melanocytic lineage. However, no gp100 protein could be detected by either Western blotting or cytotoxicity assays. Therefore, at the protein level, gp100 remains exclusive for cells of melanocytic origin despite its transcription in many cell types. The major implication of this finding is that screening of patient material for gp100 expression should preferrably be performed by antibody staining. Reverse transcriptase polymerase chain reaction (RT-PCR) can be employed, provided that it is performed in a tightly controlled, semiquantitative setting.

**Keywords:** gp100; RT-PCR; melanoma; renal cell carcinoma; tumour-associated antigens

The molecular cloning of tumour-associated antigens has provided new tools for the immunotherapy of cancer (reviewed in Van den Eynde and Brichard, 1995). Now it has become feasible to immunize cancer patients against these antigens to stimulate specifically a cellular anti-tumour response (Marchand et al, 1995). To select patients eligible for immunotherapeutic protocols, the antigenic profiles of the patients' tumours must be characterized. This is usually achieved by reverse transcriptase polymerase chain reaction (RT-PCR) using RNA obtained from tumour samples when available. An alternative source of tumour cells is whole blood, which often contains numerous circulating residual tumour cells (Brossart et al, 1995; Hoon et al, 1995). A suitable antigen to target in melanoma is gp100 because it is thought to be specific for cells of melanocytic lineage and it is adequately expressed in melanoma cells. Expression in non-melanocytic cells, at least as measured by antibody reactivity (Vennegoor et al, 1988) or Northern analysis (Kawakami et al, 1994), is virtually absent. In a routine RT-PCR screening of a number of human tumour samples and normal tissues, we noticed to our surprise that gp100 transcripts were present in almost all materials tested, whereas the protein was not detectable.

*Received 20 January 1997*
*Revised 25 April 1997*
*Accepted 12 May 1997*

*Correspondence to:* PI Schrier, Department of Clinical Oncology, University Hospital Leiden, bldg. 1, K1, PO Box 9600, 2300 RC Leiden, The Netherlands

### MATERIALS AND METHODS

### Tumour cell lines, samples and normal tissues

The renal cell carcinoma cell lines (RCC) LE-9104-RCC, LE-9211-RCC and LE-9415-RCC and the melanoma cell line Mel 603 were established in our laboratory. The RCC cell line SK-RC-7 was kindly provided by Dr E Oosterwijk (Department of Urology, Nijmegen University, The Netherlands). The RCC cell lines MZ-1851-RCC and Camejo and melanoma cell lines MZ-2-mel and MZ-7.4-mel were generously provided by Dr B Seliger (J Gutenberg University, Mainz, Germany). The breast carcinoma cell lines (BRCA) MCF-7 and SK-BR-3 were a gift from Dr R Kuypers (Department of Pathology, Leiden University, The Netherlands). The ovarian carcinoma cell lines (OVCA) COV 434 and COV 413A were established in our laboratory (Van den Berg-Bakker et al, 1993). The melanoma cell line Mel 624 and TIL 1200 lymphocytes were kindly provided by Dr Y Kawakami (NCI, NIH, Bethesda, MD, USA). LE-8915-EBV and PS-EBV are EBV-transformed B-cell lines established in our laboratory. BLM is a melanoma cell line (Bakker et al, 1994). Normal tissues were obtained after death from a woman without cancer. Fresh retina was kindly provided by Dr M Jager (Department of Ophthalmology, Leiden University, The Netherlands).

### RNA isolation and cDNA synthesis

Total RNA was isolated by guanidine-thiocyanate (HSCN) extraction as described by Chomczynski and Sacchi (1987) or using RNAzolB (Tel-Test, Friendswood, TX, USA). Before cDNA

**Table 1** Absence of cytotoxicity of TIL 1200 towards gp100-positive RCC cell lines

| | HLA-A*0201 | gp100 | % specific lysis (+ W6/32)[b] | | |
|---|---|---|---|---|---|
| E:T ratio | | | 30:1 | 10:1 | 3:1 |
| Cell line[a] | | | | | |
| BLM | + | – | 2 | 2 | 0 |
| | | | (0) | (– 2) | (– 1) |
| Mel 624 | + | + | 58 | 37 | 17 |
| | | | (14) | (7) | (0) |
| LE-9104-RCC | + | + | 2 | 0 | 0 |
| | | | (3) | (1) | (– 1) |
| LE-9415-RCC | + | + | – 2 | – 2 | – 5 |
| | | | (– 5) | (– 4) | (– 3) |
| MZ-1851-RCC | + | + | 3 | 4 | 2 |
| | | | (2) | (– 1) | (1) |
| Camejo | – | + | 0 | 0 | 0 |
| | | | (0) | (1) | (0) |

[a]Cell lines were tested in the presence of 10-fold excess cold K562. [b]The percentage of lysis after blocking with a 1:150 dilution of W6/32 is shown in brackets.

synthesis, RNA from the cell lines was treated with DNAase I (Gibco BRL, Breda, The Netherlands) for 30 min at 37°C followed by phenol extraction and precipitation. RNA isolations from the post-mortem tissues yielded such small amounts of RNA that we did not perform DNAase treatment. For cDNA synthesis, 2 μg of total RNA was reverse transcribed in first-strand buffer using 200 U of M-MLV-RT (Gibco BRL) in the presence of 40 pmol of oligo-dT(18), 10 mM of each nucleotide, 200 nmol of DTT and 20 U of RNasin (Gibco BRL) in a volume of 20 μl. After 1 h at 42°C, 80 μl of sterile water was added (Van den Eynde et al, 1995). The integrity of the cDNA was assessed by PCR for β-actin.

### PCR analysis

PCR was performed with 2.5 μl of the cDNA in 1 × PCR buffer, 2 mM magnesium chloride, dNTP (10 mM each), 10 pmol of both primers, 0.05% W1, 0.5 U of *Taq* (Gibco BRL) in a volume of 25 μl. The PCR primers and programmes used were: β-actin, sense 5′-GGCATCGTGATGGACTCCG-3′, antisense 5′-GCTG-GAAGGTGGACAGCGA-3′, 5 min at 94°C (1 min at 94°C, 2 min at 68°C, 2 min at 72°C), 30×, 15 min at 72°C (Guilloux et al, 1996). Gp100, sense 5′-TATTGAAAGTGCCGAGATCC-3′, antisense 5′-TGCAAGGACCACAGCCATC-3′, 5 min at 94°C (1 min at 94°C, 1 min at 60°C, 1 min 72°C), 35×, 15 min at 72°C (Adema et at, 1994). Tyrosinase, sense 5′-TTGGCAGATTGTCT-GTAGCC-3′, antisense 5′-AGGCATTGTGCATGCTGCTT-3′, 5 min at 94°C (1 min at 94°C, 1 min at 60°C, 1 min at 72°C), 30 ×, 15 min at 72°C (Smith et al, 1991). MART-1/MelanA, sense 5′-CTGACCCTACAAGATGCCAAGAG-3′, antisense 5′-ATCA-TGCATTGCAACATTTATTGATGGAG-3′, 5 min at 94°C (1 min at 94°C, 2 min at 63°C, 2 min at 72°C) 35 ×, 15 min at 72°C. RAGE-1, sense 5′-GTGTCTCCTTCGTCTCTACTA-3′, antisense 5′-GAGGTATTCCTGATCCTG-3′, 5 min at 94°C (1 min at 94°C, 2 min at 56°C, 3 min at 72°C), 33 ×, 15 min at 72°C (Gaugler et al, 1996). The expected sizes of the PCR products were: β-actin, 612 bp; gp100, 360 bp; tyrosinase, 284 bp; MART-1/MelanA, 603 bp; RAGE-1, 239 bp. A total of 15 μl of the PCR products was run on 2% agarose gels stained with ethidium bromide. For semi-quantitative analysis 0.2 μl of [$^{32}$P]-dCTP was added to the PCR mixture. For both gp100 and β-actin we observed that amplification was linear at 25 and 21 cycles respectively. After amplification, 10 μl of the radioactive PCR products was separated on 6% acrylamide gels and the intensities of the PCR products were analysed using a Phosphor-Imager (Molecular Dynamics, Sunnyvale, CA, USA).

**Figure 1** RT-PCR analysis of tumour-associated antigens. RT-PCR products of gp100, MART-1, RAGE-1, tyrosinase and β-actin transcripts in tumour cell lines (left) and in normal tissues (right) were detected by ethidium bromide-stained 2% agarose gels. The samples were loaded as indicated on the top of the figure. At the left, the positions of the kb marker are indicated. The upper band in the gp100 PCR indicated in the figure with an asterisk is the result of amplification of genomic DNA

© Cancer Research Campaign 1997

**Figure 2** Expression of gp100 protein in RCC cell lines as determined by Western blotting. Lanes 1–4, RCC cell lines as indicated in the figure; lane 5, COS-7 cells; lanes 6 and 7, melanoma cell lines as indicated. The numbers on the left indicate the positions of the protein molecular weight marker

**Figure 3** Levels of gp100 expression in Mel 624, MCF-7 and JY cell lines. cDNA as indicated in the figure was used in various dilutions in a radioactive RT-PCR. The PCR products of β-actin (top) or gp100 (lower) were separated on acrylamide gels and quantified by phosphor-imaging

### Western blotting

Cell lysates were prepared according to Vennegoor et al (1988). An aliquot of protein was loaded (20 μg per lane) on a 10% SDS-PAGE. Human gp100 was detected by polyclonal rabbit antiserum AZN-LAM, which was raised against the C-terminal 16-mer peptide of human gp100 protein, and which was chemically coupled to KLH (Pierce, Rockford, IL, USA). The serum was used in a 1:3000 dilution for Western blotting. For immunodetection, goat anti-rabbit-peroxidase (Zymed, San Francisco, CA, USA) and ECL (Amersham, Buckinghamshire, UK) were used (MWJS manuscript in preparation).

### Cytotoxicity assay

Cytotoxicity towards the cell lines was performed as described previously (Bakker et al, 1995). The target cells were pretreated with 50 U $ml^{-1}$ γ-IFN for 48 h before testing. Antibody blocking of cytotoxicity was performed by incubation of the chromium-labelled target cells with W6/32 ascites in 1:100 dilution at 20°C for 30 min. The final concentration of W6/32 was 1:150.

### RESULTS

### gp100 but not MART-1/MelanA or tyrosinase transcripts can be detected by RT-PCR in tumour cell lines and normal tissues

To assess tumour antigen expression by a panel of tumour cell lines including cell lines of non-melanocytic origin, we applied RT-PCR for the currently known tumour-specific and melanocyte lineage-specific antigens that may serve as targets for tumour-specific cytotoxic T cells (CTL). Unexpectedly, we found gp100 transcripts in cell lines of non-melanocytic origin, including 24/24 renal cell carcinoma cell lines, 8/8 ovarian carcinoma cell lines and 6/6 breast carcinoma cell lines. In Figure 1, the gp100-specific PCR products of a representative selection of these different cell lines are shown. In contrast, no tyrosinase or MART-1/MelanA transcripts were detected, except in cells of melanocytic origin. The RAGE-1 cDNA, which was recently cloned from a renal cell carcinoma cell line, was detected in RCC cell line MZ-1851-RCC and in fresh retina (Figure 1) (Gaugler et al, 1996).

gp100 has been reported to be specific for cells of the melanocyte lineage. This was based on immunohistochemical analysis and Northern analysis but not on results from RT-PCR (Vennegoor et al, 1988; Kawakami et al, 1994). To investigate further the presence of gp100 transcripts in non-melanocytic cells, we analysed different normal tissues. Figure 1 shows that 9 out of 11 of the tested samples were positive for gp100, including seven from fresh normal tissues and two from EBV-transformed B-cell lines. The upper band that was sometimes observed in the fresh normal tissues is derived from genomic DNA contamination. In the gp100 sequence a small intron of 102 nt is present between the primers that were used for the gp100 PCR. After DNAase treatment of RNA before cDNA synthesis, the upper band is not amplified. To confirm that the amplified product was derived from gp100 transcripts, we cloned the PCR product and sequenced ten independent clones. These all represented the gp100 cDNA sequence (data not shown). In addition, another primer set specific for the 5′ end of the gp100 mRNA yielded PCR products of the expected size (not shown). In agreement with the literature, no gp100-specific hybridization could be detected by Northern blotting (not shown) (Kawakami et al, 1994).

### No gp100 protein was detected by Western blotting

Next, we analysed whether gp100 protein could be detected by Western blotting (Figure 2). In Mel 603 and MZ-7.4-mel, positive for gp100 by RT-PCR, gp100 protein was clearly detected by the AZN-LAM antiserum. In the RCC cell lines Camejo, LE-9104-RCC, SK-RC-7, LE-9211-RCC, which were all positive using RT-PCR (not shown), no gp100 protein was detected. COS-7 cells were negative for gp100 by RT-PCR and Western blotting.

### Cytotoxicity

Although we did not detect gp100 protein by Western blotting in the RCC cell lines that were all found to be positive by RT-PCR, antigenic processing of a very small amount of gp100 protein might still lead to MHC class I-mediated presentation of gp100 peptides. As CTL recognition requires only a few antigenic complexes on the cell surface, we tested a number of HLA-A*0201-positive, gp100-positive RCC cell lines for lysis using the gp100-specific TIL 1200 cytotoxic T-cell line (Bakker et al, 1995). None of the HLA-A*0201-positive, gp100-positive RCC cell lines was lysed, whereas the control melanoma cell line Mel 624 was readily lysed, suggesting that the level of expression of gp100 occurs at an immunologically irrelevant level (Table 1).

 © Cancer Research Campaign 1997

### Low expression of gp100 transcripts in RCC

Although gp100 transcripts can be detected by RT-PCR in various tissues, the gp100 protein is apparently not detected by cytotoxic T cells. To investigate whether this is caused by low transcription levels, we used semiquantitative PCR to compare the levels of expression in the various cell lines with that of Mel 624. Radioactive PCR for either β-actin or gp100 was performed using 21 and 25 cycles of amplification respectively, and the products were run on acrylamide gels and quantified by phosphor-imaging. The β-actin signals in Mel 624 and MCF-7 were similar, whereas the signal in JY cells was higher at the different cDNA dilutions (Figure 3). The gp100 signal, however, could readily be detected in the melanoma cell line even after a 1:$10^4$-fold dilution, but 1:5 dilutions of the cDNA of MCF-7 and JY cells yielded barely detectable gp100-specific PCR products.

### DISCUSSION

In this study we show that the melanocyte lineage-specific antigen gp100 can be detected by RT-PCR in tumour cell lines originating from tissues other than those of the melanocytic lineage. For the gp100 PCR, we used 35 cycles of amplification, which is similar to the number of cycles that is used for the MART-1/MelanA and RAGE-1 PCR – 35 and 33 respectively. Gp100 PCR products could even be detected with 30 cycles (not shown), indicating that gp100 transcripts are relatively easily amplified from cell lines of non-melanocytic origin. In contrast, transcription of MART-1/MelanA or tyrosinase is strictly confined to melanocyte lineage-specific cells. In normal tissues, including oesophagus, heart, kidney, liver, lymph node, ovary, retina and thyroid, gp100 was also detected by RT-PCR. Interestingly, we have not succeeded in detecting gp100 in blood from healthy donors. Using a nested PCR approach, however, Hersey et al. (personal communication) were able to detect gp100 in the blood of healthy donors.

In contrast to other antigens that were identified by expression cloning with specific T cells, the gp100 antigen was first identified by antibody reactivity (Vennegoor et al, 1988; Adema et al, 1994). Staining with MAbs NKI/beteb or HMB-45 could only be detected in cells of the melanocytic lineage. After cloning of the gp100 cDNA, the tissue-specific distribution was confirmed by Northern analysis (Kawakami et al, 1994). The gp100 protein is a transmembrane protein that localizes primarily on the inside of premelanosomal vesicles. The function of the protein is unknown at present. Using RT-PCR, we show that there is a low level of transcription of gp100 in virtually every cell type. Similar findings were published by Chelly et al (1989), who showed that various other tissue-specific genes could be detected by RT-PCR in various tissues. They suggested that modification of the chromatin structure during DNA replication allowed ubiquitous transcription factors to bind to their cognate DNA elements, resulting in a low level of transcription. The level of gp100 protein expressed, however, is undetectable in these cell types. From these findings we conclude that gp100 can still be considered as a marker for cells of melanocytic lineage.

Screening of cancer patients for the presence of tumour antigens as eligibility criteria for immunotherapeutical protocols is already performed in the case of MAGE-3 (Marchand et al, 1995). Moreover, tyrosinase-specific RT-PCR is used for the detection of micrometastases in melanoma patients (Proebstle et al, 1996; Rankin, 1996). To obtain clinically relevant information, screening for gp100 expression within tumour material should be performed by antibody staining. Our experiments suggest that lysis by gp100-specific T cells only correlates with expression of gp100 at the protein level but not with RT-PCR expression data. Alternatively, a threshold value of gp100 expression, at which CTL recognition still occurs, should be determined by semiquantitative PCR. A similar situation probably exists for the melanoma-specific antigen *N*-acetylglucosaminyl-transferase V, which could be detected by RT-PCR but not by CTL, when the antigen was expressed at levels lower than 8% of the reference (Guilloux et al, 1996). Our data indicate that a tightly controlled, semiquantitative PCR protocol should be developed in which gp100 is only detected when expressed at biologically revelant levels.

### ACKNOWLEDGEMENTS

We acknowledge Dr V Brichard (Ludwig Institute for Cancer Research, Brussels, Belgium) for providing MART-1/MelanA-specific primer sequences. We thank Ms AK Marijnissen and Ms D van Oorschot (Leiden University) for providing breast and ovarian carcinoma material. This work was supported by a grant from the Dutch Cancer Society (RUL95-1054).

### REFERENCES

Adema GJ, De Boer AJ, Vogel AM, Loenen WAM and Figdor CG (1994) Molecular characterization of the melanocyte lineage-specific antigen gp100. *J Biol Chem* **269:** 20126–20133

Bakker ABH, Schreurs MW, Tafazzul G, De Boer AJ, Kawakami Y, Adema GJ and Figdor CG (1995) Identification of a novel peptide derived from the melanocyte-specific gp100 antigen as the dominant epitope recognized by an HLA-A2.1-restricted anti-melanoma CTL line. *Int J Cancer* **62:** 97–102

Brossart P, Schmier JW, Kruger S, Willhauck M, Scheibenbogen C, Mohler T and Keilholz U (1995) A polymerase chain reaction-based semiquantitative assessment of malignant melanoma cells in peripheral blood. *Cancer Res* **55:** 4065–4068

Chelly J, Concordet J, Kaplan J and Kahn A (1989) Illegitimate transcription: transcription of any gene in any cell type. *Proc Natl Acad Sci USA* **86:** 2617–2621

Chomczynski P and Sacchi N (1987) Single-step method of RNA isolation by acid guanidinium thiocyanate-phenol-chloroform extraction. *Anal Biochem* **162:** 156–159

Gaugler B, Brouwenstijn N, Vantomme V, Szikora JP, Van der Spek CW, Patard JJ, Boon T, Schrier PI and Van den Eynde B (1996) A new gene coding for an antigen recognized by autologous CTL on a human renal carcinoma. *Immunogenetics* **44:** 323–330

Guilloux Y, Lucas S, Brichard VG, Van Pel A, Viret C, De Plaen E, Brasseur F, Lethe B, Jotereau F and Boon T (1996) A peptide recognized by human cytolytic T lymphocytes on HLA-A2 melanomas is encoded by an intron sequence of the N-acetylglucosaminyltransferase V gene. *J Exp Med* **183:** 1173–1183

Hoon DS, Wang Y, Dale PS, Conrad AJ, Schmid P, Garrison D, Kuo C, Foshag LJ, Nizze AJ and Morton DL (1995) Detection of occult melanoma cells in blood with a multiple-marker polymerase chain reaction assay. *J Clin Oncol* **13:** 2109–2116

Kawakami Y, Eliyahu S, Delgado CH, Robbins PF, Sakaguchi K, Appella E, Yannelli JR, Adema GJ, Miki T and Rosenberg SA (1994) Identification of a human melanoma antigen recognized by tumor-infiltrating lymphocytes associated with *in vivo* tumor rejection. *Proc Natl Acad Sci USA* **91:** 6458–6462

Marchand M, Weynants P, Rankin E, Arienti F, Belli F, Parmiani G, Cascinelli N, Bourlond A, Vanwijck R, Humblet Y et al (1995) Tumor regression responses in melanoma patients treated with a peptide encoded by gene MAGE-3. *Int J Cancer* **63:** 883–885

Proebstle TM, Huber R and Sterry W (1996) Detection of early micrometastases in subcutaneous fat of primary malignant melanoma patients by identification of tyrosinase mRNA. *Eur J Cancer* **32A:** 1664–1667

© Cancer Research Campaign 1997

Rankin EM (1996) Detection of early micrometastases in malignant melanoma. *Eur J Cancer* **32A:** 1627–1629

Smith B, Selby P, Southgate J, Pittman K, Bradley C and Blair GE (1991) Detection of melanoma cells in peripheral blood by means of reverse transcriptase and polymerase chain reaction. *The Lancet* **338:** 1227–1229

Van den Berg-Bakker CAM, Hagemeijer A, Franken-Postma EM, Smit VTHBM, Kuppen PJ, Van Raavenswaaij-Claasen HH, Cornelisse CJ and Schrier PI (1993) Establishment and characterization of 7 ovarian carcinoma cell lines and one granulosa tumor cell line – growth features and cytogenetics. *Int J Cancer* **53:** 613–620

Van den Eynde B, Peeters O, De Backer O, Gaugler B, Lucas S and Boon T (1995) A new family of genes coding for an antigen recognized by autologous cytolytic T lymphocytes on a human melanoma. *J Exp Med* **182:** 689–698

Van den Eynde B and Brichard VG (1995) New tumor antigens recognized by T cells. *Curr Opin Immunol* **7:** 674–681

Vennegoor C, Hageman P, Van Nouhuijs H, Ruiter DJ, Calafat J, Ringens PJ and Rumke P (1988) A monoclonal antibody specific for cells of the melanocyte lineage. *Am J Pathol* **130:** 179–192

*© Cancer Research Campaign 1997*